# Continuous growth reference from 24^th ^week of gestation to 24 months by gender

**DOI:** 10.1186/1471-2431-8-8

**Published:** 2008-02-29

**Authors:** Aimon Niklasson, Kerstin Albertsson-Wikland

**Affiliations:** 1 Göteborg Pediatric Growth Research Centre, Department of Pediatrics, the Institute for Clinical Sciences at the Sahlgrenska Academy, University of Gothenburg, Göteborg, Sweden

## Abstract

**Background:**

Growth charts and child growth assessment have become prime global instruments in child health practice over the 30 years. An updated, continuous growth standard that bridges size at birth values with postnatal growth values can improve child growth screening and monitoring.

**Methods:**

This novel growth chart was constructed from two sources of information. Size at birth (weight, length and head circumference) reference values were updated based on information of normal deliveries (i.e. singleton live births without severe congenital malformation, with healthy mothers and born vaginally) from the Swedish Medical Birth Registry, 1990–1999 (n = 810393). Weight was evaluated using logarithmic transformation as for postnatal weight. Standard deviations were estimated from data within the empirical mean ± 1.0 SD for each gestational week and gender. These values were smoothed by empirical curve-fitting together with values from our recently published postnatal growth reference including 3650 longitudinally followed children from birth to final height [9]. Timescale and weight axes were made logarithmic in order to magnify the early time part of the graph.

**Results:**

This study presents the first continuous gender specific growth chart from birth irrespective of gestational age at birth until 2 years of age for weight, length and head circumference. Birth weight at 40 weeks of gestation increased approximately 100 gram and length increased only 1 mm compared with earlier Swedish reference from 1977–81. The curve is now less S-shaped as compared with earlier curves and compared with 4 curves from other countries and with more constant variation over the whole range.

**Conclusion:**

Our values picture the unrestricted pattern of growth improving the detection of a deviating growth pattern, when the growth of an individual infant is plotted on the charts. Especially for very preterm infants age corrected growth can be more easily evaluated although it must be recognized that the early comparison is with what is estimated as normal growth in uterus. The reference values are useful in child health care systems for population screening, but also in research or in the clinic for evaluating various growth promoting interventions – either nutritional, surgical or therapeutic – that might affect a child in early life.

## Background

Growth charts and child growth assessment have become prime global instruments in child health practice over the past three decades [1]. Many cross-sectional growth reference standards have been published, for both postnatal growth as well as at birth. However, so far there is no continuous growth standard bridging size at birth values with postnatal growth values, enabling growth of an individual child to be evaluated by means of one single growth chart, rather than two separate ones. There are many reasons why such a reference standard does not exist. One explanation is the lack of nationally- representative data for one or other of the two age periods. Another reason is that most national birth growth references – including the most recent – only include birth weight, but not length or head circumference [2-5]. Largo *et al*. have presented both intra-uterine and extra-uterine growth reference values for weight, length and head circumference based on two sets of series in the same chart [6]; but the extra-uterine values were restricted to 8 weeks of age, and there was no attempt to join the two set of references into one single continuous smooth reference. A meta analysis of mean values in published growth reference studies 1980–2002 has been published, still only up to 10 weeks after full term [7] highlighting the clinical demand, not least for the neonatal units, for gender specific growth charts bridging the pre-postnatal growth period during which most catch up growth occur.

The latest updated Swedish national growth reference at birth was published in 1991, derived from the SMBR and based on the total Swedish cohort of infants born from 1977–1981 (n = 475 588), [8]. A "healthy population" (79%) was extracted and weekly (28–42 weeks) grouped data for length, weight and head circumference were presented for boys and girls, respectively. The latest updated postnatal Swedish national growth reference of height, weight and length – from birth to 18 years of age – was introduced in 2000, derived from a longitudinal study of 3 650 healthy children born in Göteborg, Sweden in 1974 [9].

The aim of the present study was to update the size at birth reference values for the total Swedish national birth cohort born between 1990 and 1999, not only for weeks 28–42 of gestation, but also for weeks 24 to 27, and to bridge these values by mathematical functions with the recently published postnatal growth reference values – for the first time producing continuous, smoothed and gender specific growth reference values from 24^th ^week of gestation to 24 months of age.

## Methods

### The Swedish birth register

The study was based on data from the SMBR, 1990 to 1999, compiled from records through standardized sets of questionnaire completed at all antenatal care clinics and delivery units in Sweden, as well as during paediatric examinations of newborn infants. It records virtually all (98–99%) births in Sweden and the quality of the data was investigated and considered satisfactory [10]. The records are linked to the Registry of Population, which includes the Death Registry.

### Postnatal longitudinal growth study

The study material and methods have been described in detail elsewhere [9,11]. Briefly, the study population comprised 5 111 pupils in the final grade of school in 1992 in Göteborg, Sweden. Of these, 174 pupils were not willing to participate, and another 449 failed to attend the investigation. Of the remaining cohort of 4 488, 76.8% were born in 1974, 16.8% in 1973, 3.6% in 1975 and 3.0% before 1973. In 1992, a special study team visited the schools. All participating children completed a health questionnaire as their weight and height were measured in a standard way, using a calibrated Harpenden stadiometer.

All health records for the children from birth until the final grade were obtained from Child Health Care Units and schools – an average of 14.4 measurements for each child. Information on parental height and weight was also obtained. Information from birth and the perinatal period was provided by the SMBR of the Swedish National Board for Health and Welfare. The group included only healthy children for whom there was information on gestational age and size at birth, and who were born at full term (37–43 weeks). A total of 529 children were excluded due to lack of birth data; half of them were not born in Sweden, 160 due to prematurity or post maturity, and another 141 due to chronic disease or medical treatment. The number of healthy full-term children who remained for the analysis, with complete longitudinal growth data for height, weight and head circumference from birth to final height, was 3 650 (1 801 girls and 1 849 boys).

The postnatal growth reference values used in this work – e.g. mean and SD values for height and weight from birth to final height – and head circumference from birth until 4 years of age have already been published elsewhere [9].

### Statistical analysis

#### Body size at birth

It is a well known phenomenon that the standard deviation (SD) for all sizes at birth follows a u-shaped pattern over the gestational ages, with the highest values at week 24–30 [2-5,8]. This phenomenon may be caused by a larger proportion of outlying observations in the lower and upper tail of the distribution for a certain body measurement in lower gestational ages than in higher ages, rather than reflecting a biological process [4,5]. One reason for this is a wrongly determined length of gestation; and such an error has a larger impact on babies born prematurely than for term babies, due to the decelerating growth rate by age. Another reason may be caused by the fact that it is more difficult to take an accurate body measurement in premature babies than with full-term babies. There may also be an inflation of the SD values because each gestational age includes babies with 7 days variation in age; the latter will in that case be true for all gestational ages. We also made an adjustment for the range of ages within a gestational age, i.e. 7 days. The impact of this adjustment was minor, giving about a 1% reduction in the SD values for the size at birth measures.

First, we deleted the information for deliveries not regarded as normal from the analysis, in order to give size at birth reference values based on a "healthy" population. For this reason we excluded deliveries with stillbirths, severe congenital malformations, multiple births, maternal diseases (urine infection, kidney disease, diabetes, epilepsy, asthma, ulcerative colities and systemic lupus erythematosis) and records with missing gender and missing gestational age, and babies delivered by caesarean section with possible over- representation of growth retarded infants.

Second, we deleted all biologically unrealistic height, weight and head circumference values, e.g. all values lying outside mean ± 6.0 SD.

Based on the "healthy" sample, we estimated the mean and SD in three different ways: (a) based on the whole sample, (b) after adjusting for the variation in age, i.e. days within a gestational week, with individual values interpolated to the age representing the mean age of the interval by using the mean functional value for the body measurement over the interval, and (c) by using the central portion of the distribution for the age adjusted values – a distribution "trimming" method used for samples including all babies lying within mean ± 0.5 SD, mean ± 0.6 SD, .... and mean ± 2.0 SD. The algorithm of the trimming method is provided in the Appendix. We also computed the distribution skewness and kurtosis values for each sample distribution to estimate if the sample was approximately normally distributed or not. The mathematical formulas are in the Appendix.

We adopted a logarithmic transformation to birth weight values, since this is the transformation used from birth to final height [9]. It has previously been reported that the birth weight distribution of Swedish newborns is positively skewed and that a transformation should preferably be applied [8]. The optimum transformation was found to be a square root transformation.

We also estimated the mean and SD for the three body measurements by pooling the observations for gestational weeks 24–26 and 27–29, respectively, due to the relatively low sample size in lower ages. The individual values were interpolated to the age representing the mean age of the interval by using the mean functional value for the body measurement over the interval.

#### Body size at birth to final height

The postnatal growth reference mean and SD values for height, weight and head circumference from birth to final height used in this study are the same as the one previously published [9].

#### Curve fitting of the mean and SD values

Both the mean and SD values were fitted from gestational week 24 to final height by means of empirical mathematical functions as smoothing procedures for the graphs, omitting values after 40 weeks of gestational age in the first data set.

## Results

### Sample selection

The Swedish Medical Birth Register includes information for 1 053 425 births between 1990 and 1999. We excluded all stillbirths (n = 3 666), severe congenital malformations (n = 38 042), multiple births (n = 29 607), missing gender (n = 226), missing gestational age (n = 2 213), maternal diseases (urine infection, kidney disease, diabetes, epilepsy, asthma, ulcerative colitis and systemic lupus erythematosis – in total n = 141 348) and babies delivered by caesarean section (n = 55721 in which growth retardation is overrepresented). We also deleted unrealistic body measurement values at birth, i.e. outside ± 6 SD from the mean (n = 865). The information for the remaining 810 393 deliveries was kept for the analysis; 415 110 boys and 395 283 girls. Finally there were some missing values among the 810 393 selected cases so the effective sample sizes are 801 641, 808 312 and 775 399 for birth length, birth weight and head circumference, respectively.

Some differences in the mean size at birth values between the previous Swedish reference [8] and the one presented here were observed, reflecting a secular change from 1977–1981 to 1991–1999. For boys born at 40 weeks of gestation, mean birth weight increased from 3640.8 g to 3735.3 g, mean birth length from 51.22 to 51.28 and mean BMI from 13.80 to 14.17. For girls born at 40 weeks of gestation, mean birth weight increased from 3493.9 g to 3603.7 g, mean birth length from 50.33 to 50.44 and mean BMI from 13.76 to 14.13. The increase in mean weight and mean BMI was 3% for both sexes, while mean length increased by 0.1–0.2%.

### Obtaining reference values

Tables 1, 2, 3 include the mean and SD values for length, weight and head circumference between 24 to 42 weeks of gestational length based on the total sample with and without adjustment – linear interpolation – for the exact age, i.e. in days rather than in weeks. The age adjusted SD values were about 1% less than the SD values computed on the total sample without an age adjustment: birth weight – boys 1.03%, girls 1.19%, all 1.11%; birth length – boys 1.30%, girls 1.03%, all 1.16%; head circumference – boys 1.12%, girls 1.12%, all 1.12%. However, the skewness and kurtosis values for the two ways of computing the reference values were similar. Virtually all kurtosis values for all three measurements and for both sexes were between 0 and 1, or close to zero – as expected for a normal distribution for a gestational age above 34 weeks. However, virtually all kurtosis values were much higher for the younger age groups.

**Table 1:** Summary statistics for length at birth by gestational age (GA) (weeks) based on raw data and adjusted data from the total population, and on the selected sample, Sweden, 1990–1999. Note: GA = 24 corresponds to 24 weeks+0 days etc as in the graphs.

	Total				Adjusted				Delta = 1.0			
			
GA	Mean	SD	SKEW	KURT	Mean	SD	SKEW	KURT	Mean	SD	SKEW	KURT
***Boys***												
24	32.19	1.69	0.24	-0.61	-0.56	1.77	0.26	-0.20	-0.71	1.88	0.14	0.05
25	33.78	1.88	0.00	0.51	-0.64	1.81	-0.06	0.23	-0.65	1.50	-0.30	0.41
26	34.77	2.31	-1.75	5.78	-1.39	2.22	-1.81	5.91	-1.19	1.76	-0.08	-0.09
*24–26*	*33.94*	*2.24*	*-0.64*	*1.52*	*-0.96*	*2.02*	*-1.15*	*4.68*	*-0.88*	*1.68*	*-0.22*	*0.09*
27	37.07	3.50	1.86	5.53	-0.52	3.46	2.06	6.51	-0.97	2.13	0.01	-0.05
28	38.08	2.73	-0.35	3.22	-0.97	2.70	-0.26	3.14	-0.86	1.91	-0.46	0.71
29	39.87	3.45	0.02	3.79	-0.60	3.45	0.04	3.82	-0.55	2.17	-0.11	0.09
*27–29*	*38.46*	*3.44*	*0.55*	*2.74*	*-0.70*	*3.22*	*0.74*	*5.06*	*-0.76*	*2.07*	*-0.15*	*0.17*
30	41.32	2.81	0.45	3.18	-0.49	2.78	0.49	3.11	-0.55	2.24	-0.16	0.18
31	42.38	2.70	-0.12	2.11	-0.65	2.69	-0.13	2.09	-0.57	2.32	-0.14	-0.02
32	43.85	2.56	-0.56	3.12	-0.36	2.54	-0.51	3.15	-0.30	2.18	0.02	0.04
33	45.07	2.29	-0.43	2.43	-0.27	2.26	-0.43	2.48	-0.21	2.11	-0.09	0.02
34	46.21	2.19	-0.34	1.62	-0.20	2.17	-0.37	1.85	-0.15	1.96	-0.06	0.05
35	47.07	2.10	-0.29	1.53	-0.36	2.08	-0.28	1.52	-0.31	1.94	-0.07	0.03
36	47.96	2.02	-0.16	0.62	-0.45	2.00	-0.16	0.62	-0.41	1.93	-0.03	0.08
37	48.90	2.01	-0.18	0.81	-0.48	1.99	-0.19	0.84	-0.44	1.89	-0.12	0.05
38	49.80	1.92	-0.09	0.74	-0.49	1.91	-0.09	0.76	-0.48	1.80	-0.05	0.19
39	50.58	1.85	-0.05	0.70	-0.57	1.84	-0.05	0.71	-0.57	1.74	0.05	0.04
40	51.28	1.84	0.04	0.41	-0.68	1.83	0.04	0.41	-0.70	1.79	0.09	-0.06
41	51.89	1.87	0.02	0.41	-0.87	1.86	0.02	0.42	-0.84	1.88	0.00	-0.05
42	52.40	1.91	0.00	0.35	-1.00	1.90	-0.03	0.65	-1.00	1.92	0.13	0.18
***Girls***												
24	32.45	3.45	3.62	18.24	-0.53	3.57	3.79	19.48	-1.02	1.95	-0.06	-0.34
25	33.88	3.99	3.23	12.96	-0.55	4.06	3.33	13.42	-1.23	1.91	0.03	0.09
26	35.15	2.53	0.98	6.88	-0.86	2.49	0.94	7.66	-0.98	1.82	0.09	0.14
*24–26*	*34.11*	*3.42*	*2.32*	*9.92*	*-0.69*	*3.29*	*3.18*	*16.26*	*-1.03*	*1.85*	*0.07*	*-0.13*
27	36.60	4.16	1.73	4.74	-0.73	4.13	1.77	5.00	-1.11	2.57	-0.20	1.00
28	37.50	2.75	0.34	2.57	-1.34	2.69	0.27	2.51	-1.35	2.35	-0.18	0.08
29	39.57	2.55	0.88	5.35	-0.66	2.49	0.88	5.28	-0.68	2.03	-0.49	0.43
*27–29*	*38.19*	*3.31*	*0.68*	*3.05*	*-0.89*	*3.03*	*1.35*	*6.31*	*-0.96*	*2.25*	*-0.22*	*0.12*
30	40.80	2.73	0.68	3.97	-0.51	2.71	0.75	4.03	-0.56	1.99	-0.33	0.14
31	42.39	2.69	0.63	1.99	-0.16	2.66	0.66	2.26	-0.25	2.14	-0.14	-0.22
32	43.24	2.52	-0.31	3.80	-0.46	2.50	-0.30	3.93	-0.41	1.98	-0.14	0.41
33	44.53	2.26	-0.24	1.66	-0.25	2.26	-0.27	1.69	-0.21	2.08	-0.08	0.03
34	45.58	2.35	-0.34	2.60	-0.22	2.35	-0.31	2.67	-0.16	2.08	-0.05	-0.05
35	46.48	2.11	-0.18	0.78	-0.31	2.09	-0.17	0.79	-0.28	2.00	0.00	-0.03
36	47.37	2.02	-0.15	0.45	-0.37	2.01	-0.14	0.42	-0.34	1.93	-0.07	-0.05
37	48.23	1.97	-0.04	0.73	-0.44	1.95	-0.03	0.73	-0.42	1.87	0.01	-0.01
38	49.03	1.89	-0.13	0.72	-0.52	1.88	-0.12	0.72	-0.49	1.81	-0.08	0.05
39	49.79	1.81	-0.07	0.75	-0.59	1.80	-0.07	0.76	-0.59	1.70	-0.08	0.15
40	50.44	1.81	-0.04	0.80	-0.71	1.80	-0.04	0.81	-0.72	1.72	0.08	0.07
41	51.01	1.83	0.03	0.54	-0.92	1.82	0.02	0.53	-0.84	1.77	0.08	0.35
42	51.44	1.85	0.10	0.41	-1.13	1.84	0.12	0.53	-1.15	1.82	0.22	0.04

**Table 2:** Summary statistics for log_10 _[weight(kg)] at birth by gestational age (GA) (weeks) based on raw data and adjusted data from the total population, and on the selected sample, Sweden, 1990–1999. Note: GA = 24 corresponds to 24 weeks+0 days etc as in the graphs.

	Total				Adjusted				Delta = 1.0			
			
GA	Mean	SD	SKEW	KURT	Mean	SD	SKEW	KURT	Mean	SD	SKEW	KURT
***Boys***												
24	-0.16	0.14	-6.14	47.15	-0.029	0.144	-6.47	50.68	-0.013	0.060	0.18	0.26
25	-0.07	0.16	0.30	23.98	-0.013	0.159	0.21	24.03	-0.022	0.053	-0.42	0.45
26	-0.03	0.08	-0.25	5.05	-0.037	0.078	-0.30	5.12	-0.031	0.056	0.04	0.15
*24–26*	*-0.07*	*0.14*	*-1.64*	*31.64*	*-0.026*	*0.128*	*-1.70*	*38.18*	*-0.024*	*0.055*	*-0.16*	*0.03*
27	0.04	0.10	1.76	9.23	-0.028	0.103	1.93	10.93	-0.029	0.058	-0.07	0.29
28	0.09	0.08	-1.41	6.08	-0.031	0.081	-1.31	6.34	-0.026	0.058	-0.11	0.24
29	0.14	0.10	-0.07	4.13	-0.038	0.100	-0.06	4.25	-0.031	0.066	-0.41	0.31
*27–29*	*0.09*	*0.11*	*0.25*	*3.72*	*-0.033*	*0.095*	*0.47*	*7.72*	*-0.028*	*0.060*	*-0.21*	*0.25*
30	0.19	0.10	0.11	3.63	-0.036	0.094	0.20	3.90	-0.033	0.064	-0.06	-0.11
31	0.24	0.09	-0.61	4.16	-0.036	0.088	-0.64	4.16	-0.030	0.063	-0.32	0.38
32	0.30	0.08	-0.28	3.58	-0.023	0.080	-0.25	3.73	-0.021	0.062	-0.12	0.00
33	0.35	0.07	-0.44	4.25	-0.015	0.074	-0.39	4.41	-0.013	0.057	-0.19	0.19
34	0.39	0.07	-0.52	2.94	-0.010	0.070	-0.54	3.23	-0.008	0.057	0.00	0.10
35	0.43	0.07	-0.58	2.33	-0.008	0.065	-0.58	2.35	-0.005	0.056	-0.10	-0.01
36	0.46	0.06	-0.42	1.29	-0.006	0.063	-0.43	1.33	-0.004	0.057	-0.10	0.02
37	0.50	0.06	-0.37	1.16	-0.004	0.060	-0.37	1.16	-0.002	0.054	-0.05	0.04
38	0.53	0.06	-0.30	0.83	-0.004	0.056	-0.29	0.84	-0.002	0.053	-0.07	0.03
39	0.55	0.05	-0.27	0.68	-0.006	0.053	-0.26	0.68	-0.005	0.051	-0.05	0.02
40	0.57	0.05	-0.21	0.45	-0.011	0.052	-0.21	0.46	-0.010	0.050	-0.04	0.02
41	0.58	0.05	-0.24	0.42	-0.019	0.052	-0.25	0.44	-0.014	0.051	-0.06	0.02
42	0.59	0.05	-0.27	0.39	-0.024	0.052	-0.22	0.40	-0.023	0.052	-0.06	0.00
***Girls***												
24	-0.15	0.10	4.20	28.58	-0.009	0.106	4.38	30.13	-0.016	0.063	-0.22	-0.06
25	-0.08	0.12	3.02	15.07	-0.006	0.122	3.20	16.26	-0.017	0.057	0.16	0.77
26	-0.05	0.08	-1.10	2.86	-0.044	0.079	-1.31	3.37	-0.036	0.055	-0.44	0.91
*24–26*	*-0.09*	*0.11*	*1.92*	*11.18*	*-0.022*	*0.104*	*2.87*	*20.82*	*-0.024*	*0.057*	*0.02*	*0.20*
27	0.02	0.14	1.75	5.95	-0.026	0.138	1.88	6.73	-0.035	0.073	-0.36	0.23
28	0.05	0.10	-0.67	1.53	-0.065	0.095	-0.73	2.21	-0.055	0.077	-0.32	0.55
29	0.13	0.08	-0.84	4.46	-0.040	0.083	-0.91	5.12	-0.034	0.064	-0.12	-0.04
*27–29*	*0.07*	*0.11*	*0.23*	*3.05*	*-0.045*	*0.105*	*0.87*	*7.90*	*-0.040*	*0.070*	*-0.20*	*0.08*
30	0.18	0.10	0.09	5.70	-0.033	0.101	0.14	6.03	-0.034	0.062	-0.12	0.03
31	0.23	0.09	0.38	2.90	-0.029	0.092	0.43	3.16	-0.029	0.063	-0.12	0.17
32	0.28	0.08	-0.25	2.56	-0.027	0.082	-0.27	2.59	-0.022	0.066	-0.25	0.08
33	0.33	0.07	-0.34	1.89	-0.019	0.073	-0.34	1.91	-0.016	0.058	-0.03	0.08
34	0.37	0.08	-0.42	1.52	-0.010	0.074	-0.41	1.64	-0.006	0.059	-0.05	0.10
35	0.41	0.07	-0.36	1.15	-0.004	0.068	-0.36	1.22	-0.001	0.060	-0.11	0.06
36	0.45	0.06	-0.31	0.96	0.000	0.064	-0.30	0.94	0.002	0.058	-0.09	0.07
37	0.48	0.06	-0.35	1.14	0.001	0.061	-0.34	1.17	0.003	0.056	-0.06	0.05
38	0.51	0.06	-0.29	0.91	0.000	0.057	-0.28	0.92	0.002	0.054	-0.04	0.03
39	0.53	0.05	-0.23	0.56	-0.003	0.053	-0.23	0.57	-0.002	0.051	-0.04	0.02
40	0.55	0.05	-0.18	0.37	-0.008	0.052	-0.17	0.38	-0.007	0.051	-0.02	0.00
41	0.57	0.05	-0.22	0.46	-0.016	0.052	-0.22	0.45	-0.011	0.051	-0.06	0.03
42	0.57	0.05	-0.19	0.29	-0.024	0.052	-0.13	0.35	-0.023	0.051	-0.01	0.01

**Table 3:** Summary statistics for head circumference at birth by gestational age (GA) (weeks) based on raw data and adjusted data from the total population, and on the selected sample, Sweden, 1990–1999. Note: GA = 24 corresponds to 24 weeks+0 days etc as in the graphs.

	Total				Adjusted				Delta = 1.0			
			
GA	Mean	SD	SKEW	KURT	Mean	SD	SKEW	KURT	Mean	SD	SKEW	KURT
***Boys***												
24	22.25	0.93	-0.24	0.19	-0.75	0.91	-0.36	0.10	-0.75	0.82	0.08	-0.46
25	23.26	1.34	0.71	0.64	-0.82	1.29	0.89	0.60	-1.02	1.12	0.54	0.27
26	24.58	1.35	-0.01	-0.22	-0.67	1.32	0.06	-0.25	-0.70	1.39	0.07	-0.37
*24–26*	*23.72*	*1.57*	*0.26*	*-0.43*	*-0.73*	*1.24*	*0.32*	*0.07*	*-0.79*	*1.21*	*0.20*	*-0.17*
27	26.05	2.49	1.89	5.38	-0.17	2.46	2.06	6.33	-0.52	1.59	0.17	0.27
28	26.71	1.72	1.26	4.74	-0.52	1.70	1.57	5.87	-0.67	1.19	-0.12	0.06
29	27.85	2.04	1.27	3.53	-0.35	2.04	1.30	3.44	-0.53	1.55	0.16	-0.24
*27–29*	*26.94*	*2.23*	*1.22*	*3.37*	*-0.35*	*2.08*	*1.79*	*6.02*	*-0.57*	*1.42*	*0.09*	*-0.06*
30	28.84	1.98	0.76	2.93	-0.29	1.95	0.86	3.24	-0.37	1.64	-0.20	0.04
31	29.57	1.73	0.21	2.99	-0.42	1.72	0.21	2.90	-0.40	1.45	-0.07	0.34
32	30.58	1.61	0.59	2.83	-0.22	1.58	0.66	3.10	-0.26	1.43	0.17	0.18
33	31.51	1.50	-0.02	1.15	-0.08	1.47	0.03	1.25	-0.10	1.45	0.00	0.24
34	32.29	1.55	-0.04	1.72	-0.04	1.53	-0.06	1.65	-0.04	1.44	-0.06	0.10
35	32.97	1.49	-0.04	0.38	-0.07	1.47	-0.04	0.42	-0.06	1.43	0.00	-0.25
36	33.57	1.45	-0.01	0.31	-0.14	1.44	-0.01	0.29	-0.13	1.45	-0.03	0.27
37	34.07	1.40	0.03	0.43	-0.28	1.40	0.03	0.43	-0.30	1.35	-0.04	-0.09
38	34.51	1.36	0.03	0.22	-0.45	1.36	0.04	0.24	-0.45	1.37	0.05	0.41
39	34.89	1.32	-0.03	0.31	-0.62	1.32	-0.02	0.32	-0.61	1.31	0.08	-0.12
40	35.32	1.32	-0.03	0.24	-0.71	1.32	-0.04	0.25	-0.72	1.31	-0.20	0.21
41	35.71	1.35	-0.07	0.25	-0.83	1.35	-0.07	0.26	-0.81	1.34	0.07	0.31
42	36.05	1.36	-0.11	0.36	-0.91	1.35	-0.10	0.35	-0.90	1.36	0.08	-0.14
***Girls***												
24	22.74	3.43	2.74	8.08	-0.47	3.58	2.74	8.08	-1.44	1.58	0.63	1.70
25	24.03	3.53	2.42	5.46	-0.12	3.57	2.45	5.59	-1.21	1.36	1.08	2.06
26	24.90	3.93	5.48	34.79	-0.35	3.88	5.50	34.89	-1.02	1.18	0.35	0.73
*24–26*	*24.12*	*3.77*	*3.87*	*21.55*	*-0.31*	*3.69*	*4.04*	*20.90*	*-1.14*	*1.34*	*0.57*	*1.01*
27	25.49	2.88	2.36	6.35	-0.63	2.86	2.42	6.63	-1.14	1.53	-0.08	-0.08
28	25.97	1.59	0.57	1.05	-1.14	1.53	0.47	0.87	-1.22	1.50	0.08	-0.03
29	27.46	1.83	1.18	3.11	-0.58	1.79	1.31	3.68	-0.74	1.52	0.04	0.23
*27–29*	*26.50*	*2.25*	*1.26*	*3.53*	*-0.77*	*2.05*	*2.11*	*8.16*	*-0.97*	*1.52*	*0.07*	*0.02*
30	28.39	1.98	0.95	2.27	-0.40	1.96	1.03	2.62	-0.56	1.37	-0.47	0.29
31	29.28	1.73	0.60	2.01	-0.33	1.71	0.60	2.27	-0.43	1.41	-0.08	0.13
32	30.10	1.63	0.07	0.92	-0.30	1.62	0.06	0.81	-0.30	1.51	-0.10	0.41
33	30.91	1.58	0.46	2.29	-0.20	1.57	0.47	2.32	-0.24	1.36	0.08	0.04
34	31.87	1.56	0.14	0.98	0.08	1.54	0.15	1.06	0.08	1.39	0.01	-0.02
35	32.55	1.48	0.20	0.87	0.09	1.46	0.21	0.92	0.09	1.42	0.00	0.32
36	33.09	1.40	0.05	0.46	0.01	1.40	0.05	0.42	0.00	1.36	-0.12	-0.14
37	33.54	1.36	0.05	0.32	-0.14	1.35	0.06	0.31	-0.14	1.35	0.02	0.39
38	33.94	1.32	0.07	0.31	-0.30	1.32	0.08	0.31	-0.31	1.31	0.05	-0.06
39	34.28	1.27	0.04	0.33	-0.49	1.26	0.05	0.33	-0.51	1.23	-0.23	0.08
40	34.68	1.26	0.02	0.25	-0.58	1.26	0.01	0.27	-0.57	1.24	0.27	0.14
41	35.04	1.28	-0.01	0.27	-0.69	1.28	-0.02	0.27	-0.69	1.31	-0.04	-0.11
42	35.32	1.30	-0.04	0.10	-0.79	1.29	-0.03	0.16	-0.83	1.33	-0.38	0.07

**Figure 1:**
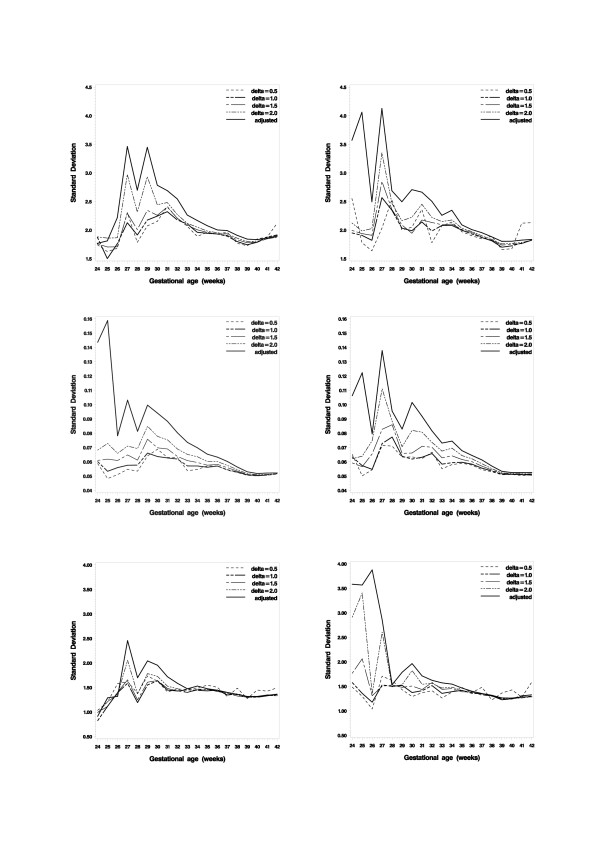
**The estimated standard deviation (SD) of body measurements at birth by gestational age (weeks).** Five different sets of SD values are shown in each graph, i.e. based on the raw data adjusted for the exact gestational age in days without any distribution trimming and for four differently trimmed series, i.e. mean ± 0.5*SD (30.85% to 69.15%), mean ± 1.0*SD (15.87% to 84.13%), mean ± 1.5*SD (6.68% to 93.32%) and mean ± 2.0*SD (2.28% to 97.73%), respectively. Graphs of the left column are for boys while those of the right column are for girls. The upper panel shows the length at birth, the middle panel shows the weight at birth and the lower panel shows the head circumference at birth.

We estimated the mean, SD, skewness and kurtosis for all three measurements and both sexes separately using the central part of each distribution sample, by trimming the tails of the distribution; within mean ± 0.5 SD, mean ± 0.6 SD, and mean ± 2.0 SD. Figure 1 shows the impact of the trimming operation in the estimation of the SD values for four different trimming situations, e.g. mean ± 0.5 SD, mean ± 1.0 SD, mean ± 1.5 SD and mean ± 2.0 SD; and they are contrasted with the SD values estimated from the non-trimmed but age-adjusted samples. The effect of estimating the SD from the central part of the distribution is clear, especially for gestational weeks below 33, and the SD values are now close to constant over the full range of gestational ages. Tables 1, 2, 3 include the skewness and kurtosis values based on the mean ± 1.0 SD trimmed samples; both values are now close to zero, i.e. the one expected for a normal distribution. To secure a more robust estimate for the mean and SD, the series from weeks 24, 25 and 26 and weeks 27, 28 and 29 were pooled, respectively. The results are also provided in Tables 1, 2, 3.

**Figure 2:**
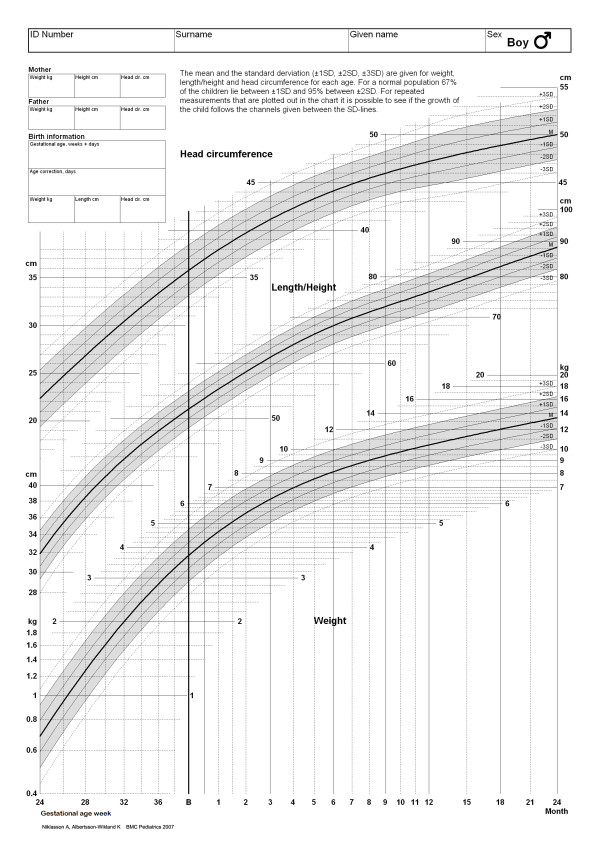
**The figure gives the new continuous smoothed Swedish growth charts from 24^th ^week of gestation to 24 months of age for boys.** Note: For a child born preterm age correction needs to be done during the remaining two years period. Note also that GA = 24 corresponds to 24 weeks+0 days etc.

**Figure 3:**
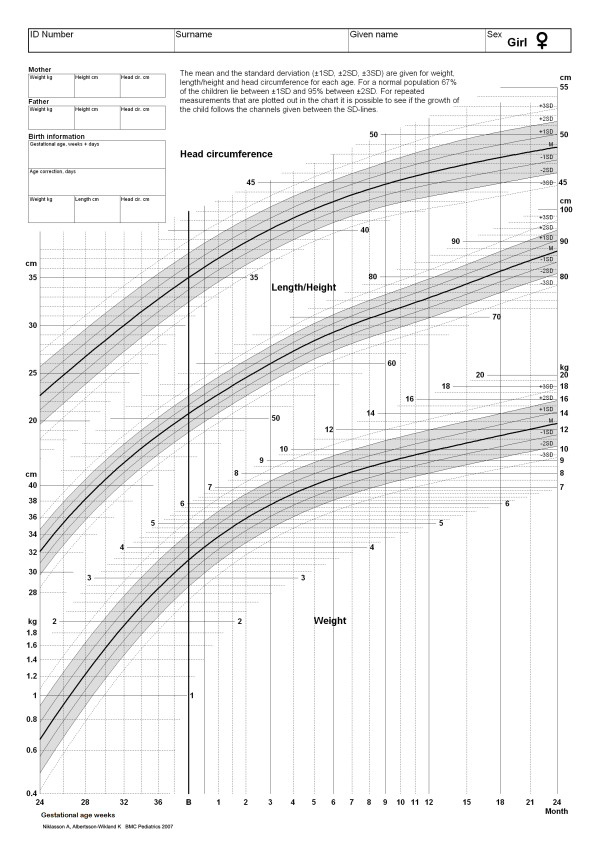
**The figure gives the new continuous smoothed Swedish growth charts from 24^th ^week of gestation to 24 months of age for girls.** Note: For a child born preterm age correction needs to be done during the remaining two years period. Note also that GA = 24 corresponds to 24 weeks+0 days etc.

For both boys and girls, the curve fitting for mean values from 24 weeks of gestation to 24 months of age gave R-square values of 1.00, 1.00 and 1.00 for height, weight and head circumference, respectively. The corresponding R-square values for SD were 0.99 for both sexes, 0.80 for boys and 0.83 for girls, and 0.35 for boys and 0.60 for girls. We could not note any systematic age-dependent bias of the residuals. Table 4 gives the smoothed mean and SD values from 24 weeks of gestation to 24 months of age, and the growth charts produced in this way are in Figure 2 for boys and Figure 3 for girls.

**Table 4:** Smoothed values of mean and standard deviation (SD) for length, weight and head circumference from birth to 2 years of age. Note: GA = 24 corresponds to 24 weeks+0 days etc as in the graphs.

	Length (cm)				Log_10 _[weight(kg)]				Head circumference (cm)			
			
	Boys		Girls		Boys		Girls		Boys		Girls	
						
Age	Mean	SD	Mean	SD	Mean	SD	Mean	SD	Mean	SD	Mean	SD
***Gestational age (weeks)***												
24	31.9	1.3	32.1	1.2	-0.165	0.064	-0.178	0.069	22.4	1.5	22.7	1.5
25	33.7	1.3	33.8	1.2	-0.092	0.063	-0.105	0.067	23.6	1.5	23.7	1.5
26	35.4	1.3	35.3	1.3	-0.023	0.062	-0.036	0.066	24.7	1.5	24.8	1.5
27	37.0	1.3	36.8	1.3	0.041	0.061	0.027	0.065	25.8	1.5	25.8	1.5
28	38.5	1.4	38.2	1.3	0.100	0.061	0.086	0.064	26.8	1.5	26.7	1.4
29	39.9	1.4	39.6	1.3	0.156	0.060	0.141	0.063	27.8	1.4	27.6	1.4
30	41.2	1.4	40.8	1.3	0.207	0.059	0.193	0.062	28.7	1.4	28.5	1.4
31	42.5	1.4	42.0	1.4	0.256	0.058	0.241	0.061	29.6	1.4	29.3	1.4
32	43.7	1.4	43.2	1.4	0.301	0.058	0.285	0.060	30.4	1.4	30.0	1.4
33	44.8	1.4	44.3	1.4	0.343	0.057	0.327	0.059	31.2	1.4	30.8	1.4
34	45.9	1.5	45.3	1.4	0.382	0.056	0.366	0.058	32.0	1.4	31.5	1.4
35	47.0	1.5	46.3	1.4	0.419	0.056	0.402	0.057	32.7	1.4	32.1	1.3
36	48.0	1.5	47.3	1.5	0.453	0.055	0.436	0.057	33.4	1.4	32.8	1.3
37	48.9	1.5	48.2	1.5	0.485	0.055	0.468	0.056	34.0	1.4	33.4	1.3
38	49.8	1.5	49.1	1.5	0.515	0.054	0.498	0.055	34.7	1.3	34.0	1.3
39	50.7	1.6	50.0	1.5	0.544	0.053	0.525	0.054	35.2	1.3	34.5	1.3
40	51.6	1.6	50.8	1.5	0.570	0.053	0.551	0.054	35.8	1.3	35.0	1.3
***Age (months)***												
3	61.3	1.8	60.0	1.8	0.798	0.048	0.775	0.048	41.1	1.2	40.1	1.2
6	68.1	2.0	66.6	2.0	0.909	0.044	0.884	0.045	44.1	1.2	43.0	1.1
9	72.5	2.2	71.0	2.2	0.974	0.042	0.947	0.044	46.0	1.1	44.8	1.1
12	76.1	2.4	74.7	2.4	1.018	0.041	0.991	0.043	47.2	1.3	46.0	1.3
15	79.6	2.6	78.2	2.6	1.053	0.041	1.026	0.043	48.1	1.4	46.9	1.3
18	82.8	2.8	81.5	2.8	1.081	0.041	1.056	0.043	48.9	1.3	47.6	1.4
21	85.7	2.9	84.5	3.0	1.106	0.042	1.082	0.043	49.5	1.3	48.2	1.3
24	88.3	3.1	87.2	3.1	1.128	0.043	1.105	0.042	50.0	1.3	48.7	1.3

### Comparison with other references

**Figure 4:**
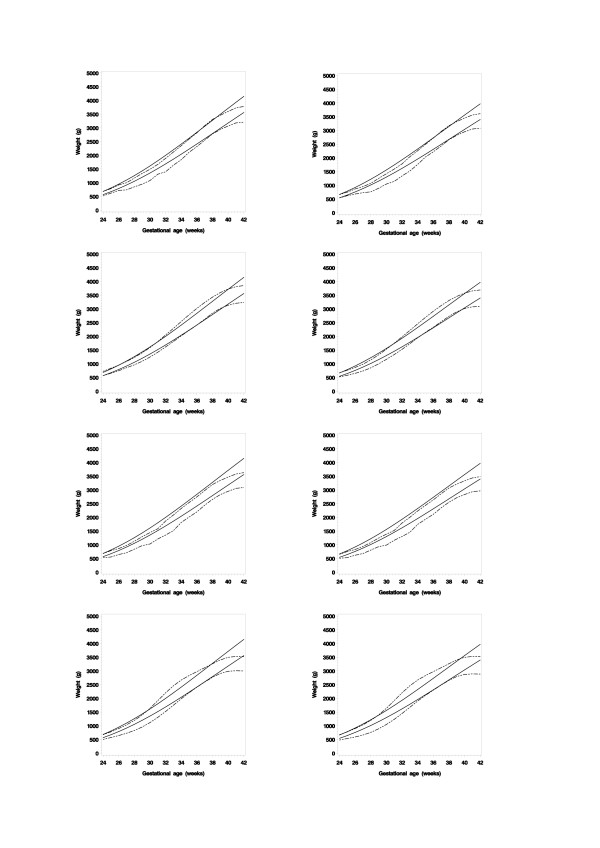
Mean and the 10^th ^centile for birth weight for boys and girls based on the present study (continues lines) compared with the same reference values for Australia, Norway, UK and US [2-5].

Figure 4 gives the mean and the 10^th ^centile for birth weight for boys and girls based on the present study; and are compared with reference values for the same range of gestational age for Australia, Norway, the UK and United States. The mean values for those four countries are falling off from about 38 weeks of gestation and the 10^th ^centile values are all lower than the Swedish corresponding values, especially in younger gestational ages.

## Discussion

To our knowledge this is the first study that gives a continuous gender specific growth standard bridging size at birth values with postnatal growth values from 24 weeks of gestational age up to 2 years of corrected age. With this, the growth of an individual child can be evaluated from birth to infancy by means of one single growth chart, rather than two separate charts. These new charts have been introduced as the Swedish national growth charts from 2006.

We were able to construct such charts because we had access to the data of two large studies – one cross-sectional at birth (n = 810393) and one longitudinal from birth to 18 years of age (n = 3650). Another important advantage was that both studies give access not only to weight, but also to length/height and head circumference. Most other studies on size at birth only include information about birth weight, not other body measurements – including recent related publications from Australia, Norway, the UK and United States [2-5] or meta-analysis using gender unspecific weight, length and head circumference up to 10 weeks postnatal age [7]. Having access to birth length may be important in some situations; for instance, it has previously been shown that birth length is a stronger risk factor for final height shortness and higher blood pressure in adulthood than birth weight [11-13].

The Swedish Medical Birth Registry has been the source of data for some 200 publications and represents one of the largest and most complete birth registers worldwide. Its great advantage is that not only birth weight, but also birth length has been recorded nationally at delivery in a standardized manner since 1973. Moreover, during the study period, gestational age has been routinely checked by ultrasound (usually in the 16^th ^week) and the quality of this data has been determined in two reports concluding it is of an acceptable quality for research purposes [10,14]. The Swedish children included in the longitudinal postnatal study were born around 1974 and raised under a relatively favourable environment for growth. We compared with cohorts born 1984 and 1990, without detecting any significant secular trend (unpublished data). Therefore, the growth patterns from this large longitudinal sample can be used to produce realistic growth reference values not only for the wider Swedish population, but also for other populations with a similar general health and socio-economic status. It is understood to be the largest longitudinal growth study spanning from birth to maturity.

We included information for 810 393 births or 76% of the total sample in the analysis. The most common reasons for exclusion were maternal diseases and caesarean section deliveries followed by severe malformation and multiple births. Only a few records (< 0.1%) were omitted due to extreme and unrealistic birth size values. We defined the "healthy" part of the populations in much the same way as other similar studies (see for instance [2-5]). The same is true for the way outlying values were identified and omitted from the analysis.

We compared the new and previous Swedish birth size values. Mean values for length are very similar for the two series, while the mean birth weight and BMI at birth increased by 3% from 1977–1981 to 1991–1999. Detail about this observation needs to be further evaluated by addressing BMI reference values in early life.

Many previous researchers have reported the specific pattern for the SD for body measurements at birth with a maximum at about 30–36 weeks of gestation [4,5,8]. This can also be noted in Figure 4 for the reference values from Australia, Norway, the UK and United States by observing the gap between the mean and the 10^th ^centile lines. It is generally believed that the "true" SD values increases with gestational age, rather than reaching a peak maximum at 30–36 weeks. Different techniques have applied to handle this gestational age SD inconsistency; either by adopting some mathematical model assuming a mixture of two statistical distributions – a true one and one represented miss-recorded values – or by simply adopting a constant coefficient of variation over all gestational ages [4,5,15]. Our trimming method approach allowed us to estimate the SD from the central part of the sample distribution, thus providing an alternative method; the statistical theory behind the calculation is described in the Appendix and it is relatively easy to apply to the data. We strongly feel that the SD values estimated by the trimmed sample method produces a much more realistic SD estimate than the SD values estimated directly from the full data; the former method producing more realistic distribution kurtosis values, an effect especially notable below 36 weeks of gestation. The kurtosis values for the ages above 35 weeks of gestation were, however, close to the one seen for a normal distribution when the full set was analyzed. The increased SD values in lower gestational ages are thus inconsistent with what is observed in the higher gestational ages and are a result of an increased distribution kurtosis, i.e. an increased length of a distribution's tails.

To smooth the weight data from 24 gestational weeks to 18 years after birth, one simple transformation is required to apply to the whole series. The optimal transformation for the birth data was the square-root [8] while that for the longitudinal data after birth was logarithm [9]. In Table 2, the skewness of birth weight data under the logarithm transformation is very small for week 33 to week 40 while generally acceptable for week 32 or below. Compared with the values transformed by square-root (not shown), the skewness for the log data is comparable for week 32 or above, while slightly larger for week 31 or below. Therefore, it is reasonable to apply logarithm to the birth data as well as the longitudinal data.

The size at birth growth reference charts presented here slightly differ from those published in other countries [2-5]. One difference is that our lower cut-off point – here illustrated as the 10^th ^centile in Figure 4 – is higher than the other references between 24 and 35 weeks of gestation, due to the different approaches in estimating the SD values. As a result, based on our growth charts, more children born prematurely will be classified as small for gestational age than with other charts. However, we feel that our SD estimates – and thus, the related cut-off point for smallness at birth – are more realistic since they follow a reasonable trend over the range of gestational ages compared with the mean values. Another noteworthy difference is the change of the birth weight curve in higher gestational ages (Figure 4). Other reference curves decelerate from about 38 weeks of gestation, most probably as a result of space limitation in uterus, i.e. the fetus has becomes too large to maintain its otherwise high growth rate. Our reference values continue without this decelerating pattern, as a result of simultaneously fitting a curve to the size at birth values and the postnatal values up to 18 years of age. We did not include the size at birth values over 40 weeks of gestation in the curve-fitting process, thus avoiding the inclusion of the striking decelerating pattern after this age. Our values can be seen to picture the unrestricted pattern of growth and as such improve the detection of a deviating growth pattern when the growth of an individual is plotted on our growth charts.

We have to stress that growth reference values at birth are all cross-sectional in nature, and may not at all describe the post-natal growth pattern of an individual infant born very preterm [16] but the deviation will probably reflect the biological impact better than if adjusted curves could be produced. This issue should be given high study priority, especially in relation to the continuous charts like the one proposed here.

## Conclusion

This study presents an updated size at birth reference values for the total Swedish national birth cohort between 1990 and 1999, and has bridged these values by mathematical functions with postnatal growth reference values – for the first time to our knowledge producing continuous and smoothed, gender specific growth reference values from 24^th ^week of gestation to 24 months of age. The reference values can be used in child health care systems for population screening, and they can also be useful in research and in the clinic for evaluating various growth promoting interventions – either nutritional, surgical or therapeutic – that might affect a child in early life especially if born preterm.

## Abbreviations

SMBR (Swedish Medical Birth Registry), SD (Standard deviation) Cm (Centimeter), Kg/g (Kilogram/gram), GA (Gestational age), Skew (Skewness), Kurt (Kurtosis).

## Competing interests

The author(s) declare that they have no competing interests.

## Authors' contributions

AN is responsible for the preparation of the birth data and the computations and KAW for the longitudinal data. Both authors contribute equally in the conceptions and the design of the study and in writing and revising the manuscript as well as giving the final approval for the manuscript to be sent for publication.

## Appendix

Assuming normality, to estimate the variance, skewness and kurtosis of the population based on the cases in the range of *μ *± *δσ *in a sample for certain values of *δ *> 0.

Without lost of generality, let *μ *= 0. Let ${v^{\prime }}_{2}=\int_{-\delta \sigma }^{+\delta \sigma }x^{2}\varphi \left(x;\sigma \right)dx$, where *φ *(*x*; *σ*) is the normal probability distribution function with mean 0 and variance *σ*^2^. It can be deduced that

$$c_{2}=\frac{{v^{\prime }}_{2}}{\sigma^{2}}=\left(-\frac{\delta \sqrt{2/\pi }}{\exp\left(\delta^{2}/2\right)}+2\Phi \left(\delta \right)-1\right),$$

where **Φ**(*x*) is the standard normal cumulative distribution function. Let ${v^{\prime }}_{3}=\int_{0}^{+\delta \sigma }x^{3}\varphi \left(x;\sigma \right)dx$ and $v_{3}=\int_{0}^{+\infty }x^{3}\varphi \left(x;\sigma \right)dx$. It can be shown that

$$c_{3}=\frac{{v^{\prime }}_{3}}{v_{3}}=\frac{1}{2}\left(2-\frac{2+\delta^{2}}{\exp\left(\delta^{2}/2\right)}\right).$$

Let ${v^{\prime }}_{4}=\int_{-\delta \sigma }^{+\delta \sigma }x^{4}\varphi \left(x;\sigma \right)dx$ and $v_{4}=\int_{-\infty }^{+\infty }x^{4}\varphi \left(x;\sigma \right)dx$. It can be shown that

$$c_{4}=\frac{{v^{\prime }}_{4}}{v_{4}}=\frac{-2\delta \left(3+\delta^{2}\right)+3\sqrt{2\pi }\exp\left(\delta^{2}/2\right)\left(2\Phi \left(\delta \right)-1\right)}{3\sqrt{2\pi }\exp\left(\delta^{2}/2\right)}$$

Let *p *= 2Φ(*δ*) - 1 be the proportion of observations in the range of *μ *± *δσ *under a normal distribution. Considering a sample of *n *observations, x_1_, ..., x_*n*_. Let *q*_1 _be the (1 - *p*)/2-th quantile and *q*_2 _be the (1 - (1 - *p*)/2)-th quantile and *X*_*r *_= {*x*_*i *_: *q*_1 _≤ *x*_*i *_≤ *q*_2_} be the reduced sample of size *m*. An empirical estimate of ${v^{\prime }}_{j}$, *j *= 2, 3, 4, is given as

$${{\hat{v}}^{\prime }}_{j}=\frac{1}{n-1}\sum_{X_{r}}{\left(x_{i}-\bar{x}\right)}^{j}.$$

The general sample variance, the skewness and the kurtosis are given as

$$s^{2}=\frac{1}{n-1}\sum {\left(x-\bar{x}\right)}^{2}$$

$$Skew=\frac{\frac{1}{n-1}\sum {\left(x-\bar{x}\right)}^{3}}{{\left(\frac{1}{n-1}\sum {\left(x-\bar{x}\right)}^{2}\right)}^{3/2}}$$

and

$$Kurt=\frac{\frac{1}{n-1}\sum {\left(x-\bar{x}\right)}^{4}}{{\left(\frac{1}{n-1}\sum {\left(x-\bar{x}\right)}^{2}\right)}^{2}}-3$$

respectively. Let $s_{r}^{2}$, *Skew*_*r *_and *Kurt*_*r *_be the corresponding statistics calculated based on the reduced sample. Substituting [1] – [4] into [5] – [7], we have the statistics for the full dataset evaluated based on the reduced sample as

$$s^{2}=\frac{m-1}{n-1}\cdot \frac{s_{r}^{2}}{c_{2}},$$

$$Skew={\left(\frac{n-1}{m-1}\right)}^{1/2}\frac{c_{2}^{3/2}}{c_{3}}Skew_{r},$$

and

$$Kurt=\frac{n-1}{m-1}\frac{c_{2}^{2}}{c_{4}}\left(Kurt_{r}+3\right)-3.$$

## Pre-publication history

The pre-publication history for this paper can be accessed here:


